# The COVID‐19 pandemic: agile versus blundering communication during a worldwide crisis: Important lessons for efficient communication to maintain public trust and ensure public safety

**DOI:** 10.15252/embr.202153182

**Published:** 2021-05-25

**Authors:** Gaby‐Fleur Böl

**Affiliations:** ^1^ Head of Department Risk Communication German Federal Institute for Risk Assessment Berlin Germany

## Abstract

Governments’ measures to control the COVID‐19 pandemic and public reaction hold important lessons for science and risk communication in times of crisis.

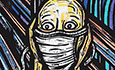

The world is in the grips of a global pandemic, the end of which is not yet in sight. Nations struggle to deal with the severe health, economic and social impacts of COVID‐19 with varying success. Their ability to handle this crisis depends on many factors, some of which, such as the availability of vaccines, are variable, while others – geographical location or population density – are determined. More importantly though, public health infrastructures, political will and action, and clear communication have so far proved to be the most successful levers for coping with the pandemic. This article examines how political will and communication in particular have helped to alleviate the impact of the virus in some countries.

… public health infrastructures, political will and action, and clear communication have so far proved to be the most successful levers for coping with the pandemic.

News that a new virus had emerged in Wuhan, China, was just of fleeting interest for most people in December 2019. This changed rapidly: by March 2020, large parts of the world had gone into lockdown to curtail the rapid spread of SARS‐CoV‐2. Many governments issued more or less harsh restrictions on private contacts, travel and other freedoms, followed by easing these regulations during the summer, which precipitated new outbreaks in the fall along with mutations of the virus that triggered new restrictions; it is likely that this pattern will continue until a sufficient number of people are vaccinated to achieve herd immunity.

COVID‐19 came “out of the blue”, hit a largely unprepared human population and has therefore affected human civilisation in an unprecedented manner (Fig 1). People are not only concerned about their health: as the pandemic continues, citizens also worry about the social, economic and psychological impacts. Even though vaccination programmes are under way, only a few countries will be able to achieve herd immunity by the summer; in the meantime, public acceptance for the ongoing restrictions of freedom are waning as the negative social and economic effects become more urgent. Thus, political action and planning along with efficient communication in particular are crucially important to ensure the public’s understanding of the situation and maintain acceptance for restrictive measure until enough vaccines become available. The antipodes in communication strategies were a mixture of evidence‐based messages, transparency, building confidence and open discussion of scientific uncertainty to gain and maintain public trust versus the unfettered spread of alternative facts, targeted disinformation and omission of important information that eventually eroded said trust.

**Figure 1:**
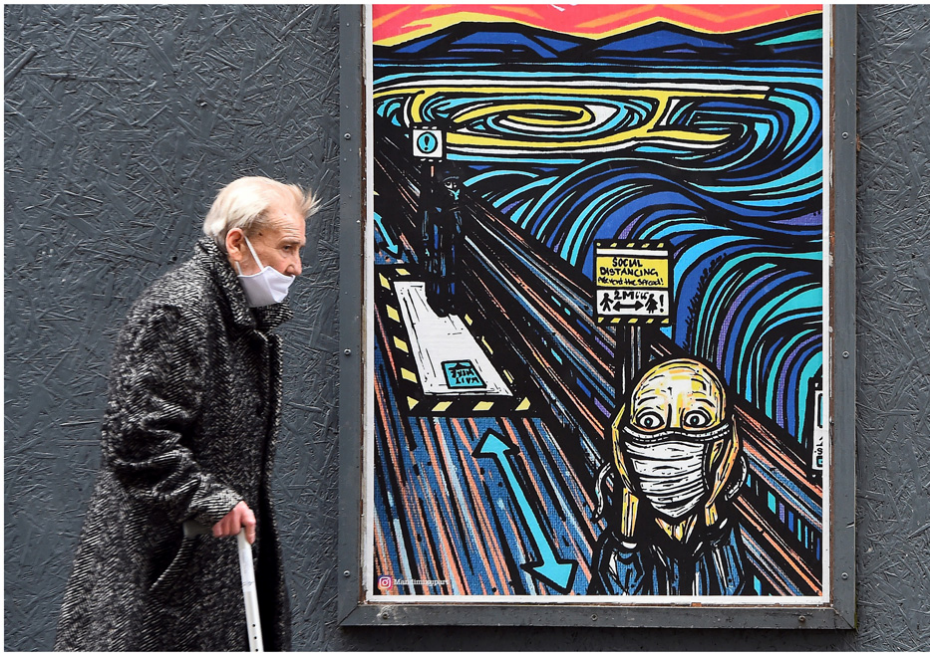
Fear in times of COVID‐19 An elderly pedestrian wearing a face mask due to the COVID‐19 pandemic, walks past graffiti depicting the subjects within famous artworks, in Glasgow on 2 September 2020 after the Scottish government imposed fresh restrictions on the city after a rise in cases of the novel coronavirus (© Andy Buchanan/AFP via Getty Images)

… political action and planning along with efficient communication in particular are crucially important to ensure the public’s understanding of the situation and maintain acceptance for restrictive measure…

## The pandemic spreads

On 31 December 2019, Chinese authorities informed the World Health Organization (WHO) about a cluster of cases of severe pneumonia of unknown cause in Wuhan City, the capital of Hubei Province in central China (WHO, 2020). On 7 January 2020, the WHO confirmed the presumption that the disease was caused by a previously unknown coronavirus which was termed SARS‐CoV‐2; the disease caused by the virus was named COVID‐19. While the COVID‐19 pandemic developed to previously unforeseen proportions, the precise origin of the outbreak remains unclear: a main suspect has been an animal market in Wuhan where bats are sold for consumption. Bats are a significant reservoir for a wide diversity of coronaviruses and 2019‐CoV is 96% identical at the whole‐genome level to a bat coronavirus (Zhou *et al,* 2020).

**Figure 2:**
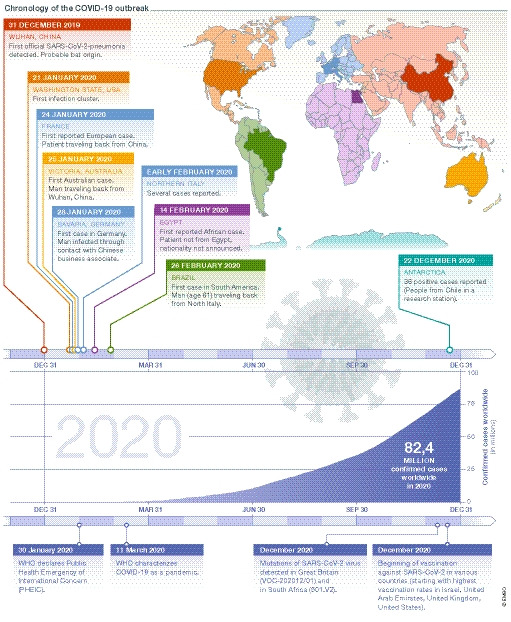
**Timeline of the COVID‐19 pandemic**.

Since, several waves of infections have hit the world (Fig 2). In Germany, the first wave took place from March to May 2020, the second began in October 2020, and Easter 21 now heralds the beginning of a third wave. Spain, Portugal, Japan and South Korea are also fighting a third wave in spring 21, while even more infectious mutations of SARS‐CoV‐2 coronavirus have emerged in the UK (B.1.1.7, known since December 2020), South Africa (B.1.351, known since August 2020), Brazil (B.1.1.248, known since December 2020) and California (CAL.20C, known since February 2021).

## Measures and policies

At the beginning of the pandemic, media coverage and guidance in the United States was rather confusing. Political assessments of the situation did not correspond with the advice of major public health institutions; in fact, US President Donald Trump was often at odds with chief scientists at the NIH or CDC. “After the President of the United States publicly urged his PR advisers to disinfect themselves by injecting disinfectant into the body, shortly afterwards the Washington State Department of Health warned citizens of the use of cleaning or disinfectant” (https://www.bbc.com/news/world‐us‐canada‐52407177). The American immunologist Anthony Fauci, Director of the National Institute of Allergy and Infectious Diseases and the White House’s health advisor, advocates precautionary measures against the spread of the virus, such as wearing masks in public: “I want to protect myself and protect others, and also because I want to make it be a symbol for people to see that that's the kind of thing you should be doing” (https://edition.cnn.com/2020/05/27/politics/fauci‐coronavirus‐wear‐masks‐cnntv/index.html). For this, he was harshly criticised by some politicians. After rapidly rising numbers of infections during the summer 2020, the United States has been running a very successful vaccination campaign since December that has met with great acceptance by the public.

Australia, which adopted a radical zero‐COVID strategy with a harsh lockdown for four months and a strict quarantine programme for travellers, has been praised for its successful handling of the pandemic: “Australia’s coronavirus lockdown strategy worked. Could this be a model for the US?” (https://edition.cnn.com/2020/09/21/australia/australia‐coronavirus‐lockdown‐intl‐hnk/index.html). As of early November 2020, the country declared itself “Covid‐free” and allowed 52,000 aspectators to watch a rugby match at Brisbane stadium. However, Australia reported new outbreaks shortly afterwards and lockdowns are constantly mandated to rein in local outbreaks. “Restrictions on our day‐to‐day lives will be in place for at least the next four weeks, with authorities working rapidly to ramp up testing and tracing systems before the rules can be relaxed”, told Australian Prime Minister Scott Morrison *The Daily Telegraph* (16 April 2020).

With a mixture of firm resolve – including border closures and consistent quarantine measures – and efficient communication, especially on social media, New Zealand’s Prime Minister Jacinda Ardern led the 4.9 million inhabitants through the pandemic as “Team New Zealand”. “In Monday’s press conference, the Prime Minister clarified that the Easter Bunny and the Tooth Fairy are essential workers delivering essential services and, as such, continue to operate throughout the alert level 4 lockdown” (*The New Zealand Herald*, 6 April 2020, https://www.nzherald.co.nz/lifestyle/COVID‐19‐coronavirus‐easter‐bunny‐and‐tooth‐fairy‐deemed‐essential‐services‐by‐jacinda‐ardern/FY5GSGW74WSSLIGSSYVA64RK7Q/). In February 2021, Prime Minister Jacinda Ardern promised “to leave no stone unturned” in the hunt for the source of the Valentine's Day COVID‐19 outbreak (*The New Zealand Herald*, 15 Febr 2021, https://www.nzherald.co.nz/nz/politics/COVID‐19‐coronavirus‐pm‐jacinda‐ardern‐vows‐to‐leave‐no‐stone‐unturned‐in‐COVID‐investigation‐first‐vaccine‐doses‐arrived‐today/ADCY6QTK5ONLZUCQOJ7D7WK2H4/). This strategy of isolation and quarantine was highly successful as New Zealand is now virtually free of the virus and has returned to normal life.

South Korea, a country with 52 million inhabitants, implemented a digital COVID‐19 tracking and management system that has been very efficient in keeping the number of cases under control. The government put the right for data privacy aside for the duration of the pandemic by mandating that citizens use a coronavirus tracking app that requires, among other things, daily measuring and reporting the body temperature. Health authorities have access to the app’s location data which allows them to track and identify infected individuals and contact persons at an early stage. Mass testing and tracing of infection chains helped to quickly localise and contain infection clusters. Intense care units were relieved and hospitals returned back to normal routine. At the beginning of the pandemic in spring 2020, South Korea was internationally praised for its handling of the COVID‐19 crisis. “We did our epidemiological investigations like police detectives. … Later, we had laws revised to prioritise social security over individual privacy at times of infectious disease crises”, said Ki Mo‐ran, an epidemiologist advising the government’s coronavirus response (*The New York Times*, 23 March 2020, https://www.nytimes.com/2020/03/23/world/asia/coronavirus‐south‐korea‐flatten‐curve.html). One year later, the situation in South Korea has changed. The number of infections is rapidly increasing, and ICU capacities are reaching breaking point.

Japan initially registered only low numbers of COVID‐19 cases and deaths compared to the rest of the world. This is all the more astonishing since the number of citizens older than 65 with a higher risk of falling seriously ill from the virus is much higher – almost twice as many as there are in Germany, for example. Geographical, climatic and cultural reasons may play a decisive role here. Japan is a group of islands, which allows for easy isolation and the warmer climatic conditions during the Japanese winter also facilitate outdoor life and easy ventilation. Wearing masks for hygienic reasons has been common in Japan long before COVID‐19. In addition, Japan has an efficient tracking system. As a result, hardly any restrictions were necessary and voluntary appeals for hygiene and distancing were sufficient. The legal situation would not have allowed curfews at that time either. However, the Japanese health system is now reaching its limits too.

However, Japan and South Korea did not begin vaccinating their population until February 2021. One reason is the comparatively high level of scepticism towards vaccines. The Japanese newspaper *Asahi* *Shimbu* published a survey on 25 January 2021, which stated that only 21% of the Japanese people would get vaccinated if a vaccine was available free of charge. Eight per cent do not want to be vaccinated at all; 70% said that they would rather wait. The Director of Duke Global Health Innovation Center, Krishna Udayakumar, commented that “The bottlenecks are really going to be on the demand side” (*The New York Times*, 31 Jan 2021, https://www.nytimes.com/2021/01/31/world/asia/japan‐south‐korea‐vaccinations.html).

Sweden has a comparatively sparse population and, at 50%, the highest proportion of single households in the EU. Sweden decided on comparatively moderate restrictions and appealing to the common sense of its citizens, a path that gained much attention internationally. However, in January 2021, Sweden was worse off than Germany in terms of COVID‐19‐related deaths, especially among the elderly. In an open letter in April 2020, around 2,000 people – including many scientists – criticised this strategy and called for more testing and a stricter course, as in neighbouring Denmark, Norway and Finland. In January 2021, the Swedish Parliament passed a new law that gave the government more options for intervention and imposing sanctions.

Whether the comparatively relaxed handling of the pandemic to achieve herd immunity – which was disputed both professionally and in the media – was a good strategy in the long term can only be conclusively judged after the pandemic. Anders Tegnell, the Chief epidemiologist at Sweden’s Public Health Agency, said: “We believe herd immunity will of course help us in the long run, and we are discussing that, but it's not like we are actively trying to achieve it… If we wanted to achieve herd immunity, we would have done nothing and let coronavirus run rampant through society” (*USA Today*, 28 April 2020, https://amp‐usatoday‐com.cdn.ampproject.org/c/s/amp.usatoday.com/amp/3031536001).

With 384 inhabitants per square kilometre, Belgium is one of the most densely populated countries in the world and recorded the highest death rate from coronavirus worldwide in May 2020 at 15.7%. According to Steven Van Gucht, a Flemish virologist and spokesperson for the Belgian National Crisis Centre, this was due to a high testing rate and counting all suspected deaths from coronavirus infections: “In most countries there were no sufficient tests and they have been underreporting the number of mortalities in retirement homes. We think that it's really important that all cases are included based on the indications we have. … We didn’t invent something new but are following the guidelines of ECDC (European Centre for Disease Prevention and Control)” (*The Brussels Times*, 24 April 2020, https://www.brusselstimes.com/news/belgium‐all‐news/107910/belgian‐expert‐confirms‐death‐rate‐and‐warns‐about‐a‐second‐wave/). The National Security Council lifted the comprehensive outdoor mask requirement at the end of September 2020, even though a second wave of COVID‐19 was imminent at that time. In terms of communication, this situation was made more difficult by the fact that Belgium did not have a new government until the beginning of October 2020, after 16 months of an interim government. Belgium still reported the highest number of coronavirus infections in the entire EU in autumn 2020. However, thanks to drastic measures, such as night curfews, mandatory mask wearing in public spaces and contact restrictions, numbers had dropped again by winter 2020/21.

Germany has adopted a range of diverse strategies to deal with the COVID‐19 pandemic owing to its federal system, which assigns public health policies to the 16 state governments. Mecklenburg‐Western Pomerania, for instance, has taken a very restrictive stance. With around 1.6 million people, the state has fewer inhabitants than Hamburg or Berlin and a very low population density of 69 inhabitants per square kilometre. Its location at the Baltic Sea makes it very popular with tourists, especially in the summer. It was therefore met with some discord when the state government denied entry to all tourists. Conversely, Thuringia, with 132 inhabitants per square kilometre, was the first state to relax contact restrictions in June 2020. More populated states, such as North Rhine‐Westphalia (526 inhabitants per square kilometre), called for relaxing restrictions based on individual epidemiological studies and supported by a PR agency. The situation is again different in Bavaria (186 inhabitants per square kilometre) which has been following a consistent approach from the start with strict regional curfews to track local outbreaks and prevent further spread. Overall, these diverse strategies have been very successful during the first wave in 2020 but the country has been struggling to control the pandemic since the fall. Moreover, overall communication to the public has been irregular and confusing with the heads of state and the federal government constantly arguing over the right course.

## Risk perception and public trust

In regard to the acceptance of restrictive measures and the resulting individual behaviour, societies’ cultural differences are a significant factor. One could categorise these countries into societies with a strong focus on individual rights, such as the United States, the UK, the Netherlands, Belgium, Denmark or Australia, and countries with a more collectivist culture, such as Japan, South Korea or Singapore (Hofstede, 1980). Thus, state measures that restrict individual rights or surveillance measures are more easily accepted by the population in some Asian countries than in the United States with its strong individualist culture.

… state measures that restrict individual rights or surveillance measures are more easily accepted by the population in some Asian countries than in the USA with its strong individualist culture.

Beyond the actual numbers of infections, it is important to take the risk perception of the population into account, not least so that crisis communication can be adapted (Cori *et al,* 2020). Criteria for maintaining public trust in government policies are, for instance, clarity and accessibility of science‐based information, active communication of scientific uncertainty, and transparency about government strategies for ongoing and planned measures (Blastland *et al,* 2020). To this end, a “COVID score” was developed to estimate how the population perceives the effectiveness of government responses in different countries (Lazarus *et al,* 2020). A study on risk perception during the early phase of the pandemic of 718 adults in the United States reported a high level of trust and low level of risk perception. Many respondents supported restrictive policies or infection prevention (McFadden *et al,* 2020).

Beyond the actual numbers of infections, it is important to take the risk perception of the population into account, not least so that crisis communication can be adapted.

Ten different countries in Europe, North America and Asia (total *n* = 6,991) have been compared to study possible predictors for risk perception regarding COVID‐19. Personal experience with the virus, cultural and social values, and trust in government as well as trust in scientific and medical experts have been analysed as critical factors (Dryhurst *et al*, 2020). It is therefore not only important for political decision‐makers to have a clear strategy in pandemic situations, but also to transparently communicate this to the public. Another analysis of nine countries and regions in Asia Pacific and Europe recommends ongoing cross‐country learning to guide future health policies, such as zero‐COVID strategies (Han *et al,* 2020). Further data about public risk perception during the pandemic are so far available for Australia (Faasse & Newby, 2020), Iran (Honarvar *et al,* 2020), and Italy and Sweden (Mondino *et al*, 2020).

Crisis preparedness also includes established networks of trust between professionals on different levels and citizens to ensure acceptance of measures that have to be adapted to actual outbreak situations in short time. Actively communicating uncertainty is also of high relevance. This was demonstrated in an online survey in Germany with a representative sample of 2,011 which showed participants four scenarios that communicated information on the future course of COVID‐19 with varying magnitudes of scientific uncertainty (Wegwarth *et al,* 2020). The largest group of respondents chose the version with the highest degree of uncertainty. This version was also the most likely to persuade people to comply with containment measures. Active communication also helps to deal with misinformation in crisis situations, especially in social media (Malecki *et al,* 2020). Better informed individual and corporate decisions depend on accessible, science‐based information and consistent public recommendations which are easily integrable into everyday life.

Better informed individual and corporate decisions depend on accessible, science‐based information and consistent public recommendations which are easily integrable into everyday life.

In addition to these international individual studies, which analysed the risk perception of the population at defined moments of the pandemic, there are so far only a few ongoing long‐term studies on risk perception. The BfR‐Corona‐Monitor is a recurring (multi‐wave) survey of the German population's perception of risks from the coronavirus (https://www.bfr.bund.de/cm/349/210413‐bfr‐corona‐monitor‐en.pdf). It includes questions on behaviour, assessment of media coverage, degree of concern, individual protective measures, assessment of state containment measures, and the controllability and probability of infection. Every week between March and May 2020, around 500 randomly selected people were contacted by phone; since June 2020, the survey continues every two weeks with about 1,000 respondents.

In March 2021, for example, about 81% of respondents said they relied on frequent ventilation to avoid contracting the virus. The mandatory use of masks was approved by 95% of respondents and the distancing regulations by 91%. In March 2021, the restrictions in day‐care centres and schools were still considered appropriate by 56%, 15 points less than before Christmas. The population is well aware that proximity to others plays a central role in the transmission of the coronavirus: around two‐thirds of the respondents still consider the probability of this pathway to be high, whereas door handles are seen by 43% as a likely route of infection. Only 11% consider transmission through food to be probable.

### Public reactions

**Figure 3:**
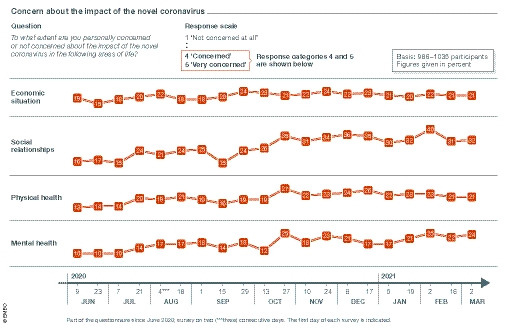
People’s concerns about the impact of the novel coronavirus on their economic and social situation, physical health and mental health Data from the BfR‐Corona‐monitor. Answers from June 2020 to March 2021 in per cent based on 986 to 1.035 participants. Values range from 1 “not concerned at all” to 5 “very concerned”

Concerns about the effects of the novel coronavirus have been growing constantly throughout the entire period, in terms of both physical and mental health, but predominantly regarding the economic situation and social connections (Fig 3). Concern about the economic situation has remained at a consistent level of about 20% since the beginning of the survey, while concerns about physical health increased from 13% at the start to 21%. People in Germany also increasingly worry about their social connections and mental health with the numbers approximately doubling between June 2020 and March 2021 in each case.

The COSMO study (COVID‐19 Snapshot Monitoring) surveys public knowledge, risk perception, protective action and trust during the current COVID‐19 outbreak (Betsch *et al*, 2020). It is based on a weekly survey of an online panel of around 1,000 people. In January 2021, the authors concluded that a certain fatigue is spreading in Germany due to, among other things, the renewed lockdown. Fifty‐five per cent of respondents reported stress; notably, people under the age of 30 consider themselves to be particularly stressed.

According to Paul Slovic’s psychometric approach to risk perception, affect and emotion play a crucial role in how people perceive news and information. In this regard, the pandemic is characterised by frequent outbreaks with high numbers of cases in, for example, retirement homes, accommodations for refugees and migrants, meat processing facilities or extended families. Media reports repeatedly convey horror scenarios that question the controllability of risk, which generates a high risk perception among the general population. Thus, transparency about the reasons for local or larger outbreaks is very important. The population should be able to realistically understand risks and become involved in dealing with the crisis process rather than being treated as mere objects. A very good example of science‐based, comprehensible communication that is highly popular in Germany is the “Coronavirus Update” podcast by the *Norddeutscher Rundfunk*. The 30‐ to 60‐minute discussions with Christian Drosten, Head of virology at Charité Hospital in Berlin, address various aspects of the SARS‐CoV‐2 virus and the COVID‐19 pandemic. In April 2020, Drosten received the Grimme Online Award for outstanding science communication during the COVID‐19 pandemic.

The population should be able to realistically understand risks and become involved in dealing with the crisis process rather than being treated as mere objects.

The current SARS‐CoV‐2 pandemic confronts governments and civil societies with dynamic situations to which they have to constantly adapt and citizens get their information from different media formats and other sources to decode the crisis. The (epidemiological) findings and forecasts on the spread of the virus are increasingly visualised in so‐called “dashboards” that combine numerical, temporal, geographical and diagrammatic into an easy‐to‐understand presentation. Dashboards visualise a large amount of data in a way that even people with less mathematical education can understand. At the beginning of the global outbreak, those dashboards – the first of which was published by Johns Hopkins University – displayed mainly the number of new cases and deaths, but this was quickly broken down into active infections/confirmed cases/deaths/recoveries. Notably, the graphic addition of the large number of those who had overcome a COVID‐19 infection changed the population’s risk perception.

## Impact of measures and vaccinations

In the wake of new, partially more infectious virus mutations, there is still no light at the end of the pandemic tunnel. Nonetheless, vaccinations begun at the end of 2020 and international frontrunner Israel announced in February 2021 that it had vaccinated about half of its population with at least one jab. Among the ten countries with the highest vaccination rate are Israel, the UK, Chile, the United States, the United Arab Emirates and Denmark.

Again, communication and media reports have made a difference in regard to public trust and risk perception of vaccines. When the German Standing Committee on Vaccination recommended AstraZeneca's COVID‐19 vaccine only for persons aged 18 to 64 years at the end of January 2021, it lowered public acceptance with people refusing the vaccine. After reports of rare cases of venous thrombosis, its use was halted for few a days in March; eventually, the Standing Committee and the German government allowed vaccinations with AstraZeneca again but this time only for people older than 64. Other countries, for instance Norway, Finland or Sweden, also temporarily stopped using the vaccine, whereas the UK decided to continue its vaccination programme. The sometimes confusing messages about the vaccine’s side effects and efficacy have probably decreased public trust in its safety.

Such comparisons between various countries demonstrate that a clear strategy along with efficient communication is an important factor for gaining public trust and acceptance whether it is for of tough lockdown measures as in Australia and New Zealand or an aggressive vaccination strategy as in Israel, the United States or the UK. The same is true for efficient tracking systems that curtail privacy rights as the examples of South Korea and Japan show. In contrast, multiple repeated short lockdown phases without clear communication or vaccination strategy are being less and less accepted by the population. Even moderate measures based mainly on recommendation as in Sweden are reaching their limits. Management strategies for coping with the crisis must be constantly adapted to meet citizens’ needs in order to achieve the broadest public acceptance.

Such comparisons between various countries demonstrate that a clear strategy along with efficient communication is an important factor for gaining public trust and acceptance.

## Mutations and new outbreaks

Regardless of the different approaches, all countries are struggling to deal with the wave‐like, partially overlapping outbreaks of COVID‐19 and the rapid spread of new mutations. Once again, it demonstrates the importance of having prepared and rehearsed communicative strategies to inform the population to overcome fears and misgivings and to explain the need for measures that interfere deeply with individual rights in order to control new outbreaks. Transparency, communicating scientific uncertainties and effective visualisation of epidemiological data are all key to gain public acceptance and support. The higher the collectivist orientation of the population, the easier this task. While countries around the world can easily seal themselves off owing to their geographical location, most nations will have to rely on popular support for the emergency measures until the vaccination campaigns ease the pressure on public and emergency health systems.

Transparency, communicating scientific uncertainties and effective visualisation of epidemiological data are all key to gain public acceptance and support.

Handling the COVID‐19 pandemic depends on many factors from health, politics and communication that can be influenced to varying degrees (Table 1). Regarding communication, the success factors include credibility of experts, institutions and decision‐makers, transparency, communication of scientific uncertainty, preparedness through established communication channels, and responsiveness to the concerns and risk perception of the population. Which factors in which country will ultimately be the deciding factor in coping with the pandemic remains to be seen but the various approaches of risk communication and their effectiveness are clearly a fascinating subject for further analysis.

**Table 1:** Relevant parameters for coping with pandemics

Given	Partly flexible	Flexible
Geographical location (option for travel control measure)	Robustness of the health system and intensive care capacity	Infection control legislation to restrict individual freedoms
Population density	Intercultural decision‐making of the population	International travel activity
Socio‐demographic structure (age, household type, education)	Credibility of experts, institutions and policy makers	Testing and tracking capacities
Climate (reduced risk of infection outdoors)	Being prepared in terms of rehearsed communication channels	Vaccine availability
	Population’s acceptance of vaccines	Risk perception of the population
		Transparency, communication of scientific uncertainty
